# Delayed intracardial shunting and hypoxemia after massive pulmonary embolism in a patient with a biventricular assist device

**DOI:** 10.1186/1749-8090-6-133

**Published:** 2011-10-11

**Authors:** Thomas Weig, Michael E Dolch, Lorenz Frey, Dirk Bruegger, Peter Boekstegers, Ralf Sodian, Michael Irlbeck

**Affiliations:** 1 Department of Anaesthesiology, Ludwig-Maximilians-University, Munich, Germany; 2 Department of Cardiovascular Surgery, Ludwig-Maximilians-University, Munich, Germany; 3 Department of Cardiology, Helios Klinikum Siegburg, Siegburg, Germany

**Keywords:** patent foramen ovale, hypoxemia, pulmonary embolism, ventricle-assist device, heart transplantation, septal occluder device

## Abstract

We describe the interdisciplinary management of a 34-year-old woman with dilated cardiomyopathy three months postpartum on a cardiac biventricular assist device (BVAD) as bridge to heart transplantation with delayed onset of intracardial shunting and subsequent hypoxemia due to massive pulmonary embolism. After emergency surgical embolectomy pulmonary function was highly compromised (PaO_2_/FiO_2 _54) requiring bifemoral veno-venous extracorporeal membrane oxygenation. Transesophageal echocardiography detected atrial level hypoxemic right-to-left shunting through a patent foramen ovale (PFO). Percutaneous closure of the PFO was achieved with a PFO occluder device. After placing the PFO occluder device oxygenation increased significantly (Δ p_a_O_2 _119 Torr). The patient received heart transplantation 20 weeks after BVAD implantation and was discharged from ICU 3 weeks after transplantation.

An increase in pulmonary vascular resistance in patients on BVAD can reopen a PFO resulting in atrial right-to-left shunting and subsequent hypoxemia. The case demonstrates the usefulness of transesophageal echocardiography examinations in the detection of this unexpected event. Percutaneous placement of a PFO occluder device is an appropriate strategy to stop intracardiac shunting through PFO in fixed elevation of pulmonary vascular resistance.

## Background

In a literature review, few cases of atrial level right-to-left shunt in patients with left ventricular assist devices are described. All these cases were detected either intraoperatively [1-3] or within the first postoperative days [4-7]. We describe a case of delayed onset of atrial level right-to-left shunt after massive pulmonary embolism on biventricular assist device (BVAD) support.

### Case Presentation

A 34 year old female patient was admitted to our hospital with dilated cardiomyopathy three months after birth of her third child. She had a known history of familial dilated cardiomyopathy. Recompensation was not achieved despite maximum medical therapy and insertion of an intra-aortic balloon pump. BVAD [Excor, Berlin Heart, Berlin, Germany] was implanted using a bi-atrial cannulation technique as bridge to heart transplantation. Perioperative transesophageal echocardiography did not show a patent foramen ovale (PFO). Postoperative recovery was immediate and the patient was discharged from the ICU on the third postoperative day.

**Figure 1:**
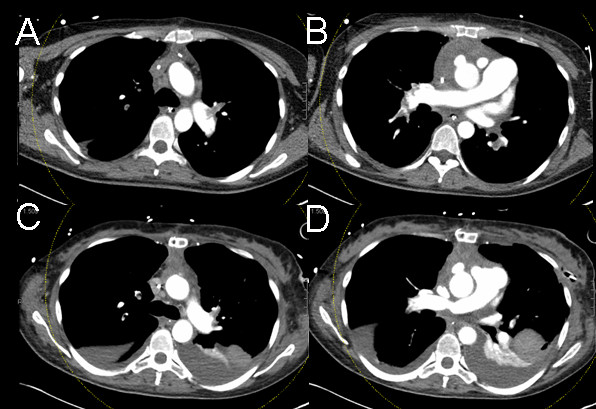
**CT-Scan: A & B before surgical embolectomy**. C & D directly after surgical embolectomy.

**Figure 2:**
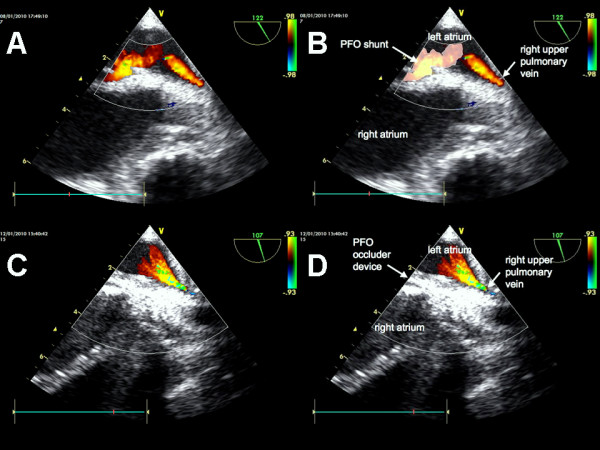
**Transesophageal echocardiography: A & B before, C & D after patent foramen ovale closure with a PFO occluder device [Amplatzer PFO Occluder^®^, AGA Medical, Plymouth, USA]**.

Four weeks after device implantation the patient developed fulminant pulmonary embolism despite therapeutic anticoagulation. Emergency surgical embolectomy for massive pulmonary embolism was performed since thrombolysis was not an option after recent implantation of an artificial heart (Figure 1). Pulmonary function was highly compromised after embolectomy and veno-venous extracorporeal membrane oxygenation (ECMO) [Bio-Console, Medtronic, Minneapolis, USA] was established using a bifemoral venous access. Weaning from veno-venous ECMO was achieved over the following week but after removal oxygenation failure reoccurred. F_i_O_2 _of 1.0 was necessary to achieve sufficient oxygen saturation (p_a_O_2_/F_i_O_2 _54). Modification of ventilator setting with adjustments of PEEP and peak inspiratory pressure did not lastingly improve oxygenation. Transesophageal echocardiography detected atrial level intracardial shunting (Figure 2). There was no improvement after application of inhaled pulmonary vasodilatators. CT-scan after surgical embolectomy showed residual emboli in the pulmonary vascular system. Invasive procedures such as re-embolectomy, topical thrombolysis or catheter fragmentation were considered as too harmful or not effective. Since right heart function was secured even with high pulmonary vascular resistance, percutaneous placement of a PFO occluder device [Amplatzer PFO Occluder^®^, AGA Medical, Plymouth, USA] was performed (Figure 2, Additional file 1). Oxygenation increased significantly after placement without change of respirator settings (Δ p_a_O_2 _119 Torr). Weaning from mechanical ventilation was successful after 15 weeks.

After 5 weeks of therapeutic anticoagulation the residual emboli diminished and pulmonary vascular resistance was measured at 184 dyne•s/cm^5 ^with activated assist device and 160 dyne•s/cm^5 ^with deactivated assist device.

Heart transplantation was performed 20 weeks after implantation of the BVAD and 16 weeks after pulmonary embolism and placement of the PFO occluder device. Discharge from ICU was 3 weeks after transplantation. Informed consent for publication was obtained from the patient.

## Discussion

The problem with PFO and left ventricular assist device leading to atrial level right-to-left shunt with consecutive hypoxemia is well described [1-7]. PFO has an incidence up to 27% in normal healthy adults as well as in adult cardiac surgical patients [8,9]. If left ventricular assist device (LVAD) is activated, left atrial unloading leads to a decrease in left atrial pressure [10]. Right atrial pressure exceeds left atrial pressure and with PFO atrial level right-to-left shunt occurs. Depending on the shunt fraction hypoxemia may occur [11].

Therefore, intraoperative transesophageal echocardiography with colour Doppler imaging and contrast with agitated saline is highly recommended before cardiopulmonary bypass and after LVAD activation [12,13]. Alternatively, manual occlusion of the pulmonary artery shortly before activation of the LVAD by the surgeon and transesophageal echocardiography studies as described are performed [14]. If PFO is detected before weaning from cardiopulmonary bypass, immediate operative closure is recommended. If shunting is detected after weaning from cardiopulmonary bypass, delayed interventional closure after stabilization is preferred if oxygenation failure is tolerable, since failure of the right heart in LVAD implantation or bleeding complications due to coagulopathy after reapplied bypass can deteriorate outcome [2]. PFO closure improved oxygenation in all known cases as it did in our patient.

There is only one other case of delayed onset of atrial level right-to-left shunt in patients on ventricular assist device [15]. In this case report, atrial level right-to-left shunt with hypoxemia occurred after replacement of the valves of a LVAD [LVAS, Novacor, Salt Lake City, USA] which had been implanted one year before. The management consisted of reduction of right atrial pressure by conservative means.

Persisting elevation of right atrial pressure due to persisting change of the pulmonary vascular resistance in a patient with a BVAD has not been described. An etiologic reason for persisting elevation of pulmonary vascular resistance can be massive pulmonary embolism as described in our case. Our report is the first description of a patient surviving massive pulmonary embolism while on BVAD, followed by successful orthotopic heart transplantation. To the best of our knowledge there is only one other published case of pulmonary embolism in a patient with a BVAD. This patient died shortly after the event [16].

Emergency surgical embolectomy is recommended in hemodynamic unstable patients with massive pulmonary embolism in a facility with cardiac surgical capabilities [17]. Catheter embolectomy should be performed in absence of cardiothoracic surgical backup [17]. In our case, thrombolysis was contraindicated. Therefore emergency surgical embolectomy was the treatment of choice. The reported median reduction of pulmonary vascular resistance achieved by surgical embolectomy is from 893 ± 443.5 dyne•s/cm^5 ^to 285 ± 214 dyne•s/cm^5 ^[18], a result that was achieved in our patient.

With regard to the planned heart transplantation, chronic thromboembolic pulmonary hypertension would have been an exclusion criterion.

## Conclusion

Diagnostic transesophageal echocardiography must be performed with relevant change in the hemodynamic situation and recurring hypoxemia in patients with VAD since increase in pulmonary vascular resistance can reopen PFO resulting in atrial level right-to-left shunting and consecutive hypoxemia.

## Consent

Written informed consent was obtained from the patient for publication of this Case report and any accompanying images. A copy of the written consent is available for review by the Editor-in-Chief of this journal.

## Competing interests

The authors declare that they have no competing interests.

## Authors' contributions

TW reviewed the case, conducted a review of the literature and drafted the manuscript. TW and MI performed the echocardiographic studies and participated in the design of the case report. RS and PB performed the operation and intervention described. MD, LF and DB confirmed the patient's diagnosis and revised the manuscript, contributing important intellectual content. All authors read and approved the final manuscript.

## Supplementary Material

Additional file 1 **Transesophageal echocardiogram**. Transesophageal echocardiogram before and after patent foramen ovale closure with a PFO occluder device [Amplatzer PFO Occluder^®^, AGA Medical, Plymouth, USA]. Click here for file
